# FT4/FT3 ratio: A novel biomarker predicts coronary microvascular dysfunction (CMD) in euthyroid INOCA patients

**DOI:** 10.3389/fendo.2022.1021326

**Published:** 2022-09-15

**Authors:** Han Zhang, Wenliang Che, Kuangyu Shi, Yan Huang, Chong Xu, Mengyu Fei, Xin Fan, Jiajia Zhang, Xueping Hu, Fan Hu, Shanshan Qin, Xiaoying Zhang, Qingqing Huang, Fei Yu

**Affiliations:** ^1^Department of Nuclear Medicine, Shanghai Tenth People’s Hospital, Tongji University School of Medicine, Shanghai, China; ^2^Institute of Nuclear Medicine, Tongji University School of Medicine, Shanghai, China; ^3^Department of Cardiology, Shanghai Tenth People’s Hospital, Tongji University School of Medicine, Shanghai, China; ^4^Department of Nuclear Medicine, University of Bern, Bern, Switzerland; ^5^Department of Informatics, Technical University of Munich, Munich, Germany; ^6^Department of Radiology, Shanghai Tenth People’s Hospital, Tongji University School of Medicine, Shanghai, China; ^7^Shanghai Key Laboratory of Molecular Imaging, Shanghai University of Medicine and Health Sciences, Shanghai, China

**Keywords:** INOCA, CMD, thyroid hormone, D-SPECT, CFR

## Abstract

**Background:**

Ischemia and no obstructive coronary artery disease (INOCA) patients who presented coronary microvascular dysfunction (CMD) demonstrate a poor prognosis, yet the risk factors for CMD remain unclear. Subtle changes in thyroid hormone levels within the normal range, especially the free thyroxine (FT4)/free triiodothyronine (FT3) ratio, have been shown to regulate the cardiovascular system. This prospective study investigated the correlation between FT4/FT3 ratio and CMD in euthyroid patients with INOCA.

**Methods:**

This prospective study (www.chictr.org.cn/, ChiCTR2000037112) recruited patients with myocardial ischemia symptoms who underwent both coronary angiography (CAG) and myocardial perfusion imaging (MPI) with dynamic single-photon emission computed tomography (D-SPECT). INOCA was defined as coronary stenosis< 50% and CMD was defined as coronary flow reserve (CFR)<2.5. All patients were excluded from abnormal thyroid function and thyroid disease history.

**Results:**

Among 71 INOCA patients (15 [21.1%] CMD), FT4 and FT4/FT3 ratio in CMD group were significantly higher and both showed significantly moderate correlation with CFR (r=-0.25, p=0.03; r=-0.34, p=0.003, respectively). The ROC curve revealed that FT4/FT3 ratio had the highest efficacy for predicting CMD with an optimized cutoff value>3.39 (AUC 0.78, p<0.001, sensitivity, 80.0%; specificity, 71.4%). Multivariate logistic regression showed that FT4/FT3 ratio was an independent predictor of CMD (OR 7.62, 95% CI 1.12-51.89, p=0.038, P for trend=0.006).

**Conclusion:**

In euthyroid INOCA patients, increased FT4/FT3 ratio levels are associated with the occurrence of CMD, presenting a novel biomarker for improving the risk stratification.

## Introduction

More than 50% of patients with myocardial ischemia symptoms who underwent coronary angiography (CAG) did not find an obstructive lesion (coronary stenosis >50%), and these patients are now diagnosed with ischemia and no obstructive coronary artery disease (INOCA) (1, 2). It was once considered a benign disease, conversely, recent studies have shown that this group of patients has a high rate of mortality and adverse clinical outcomes (3). Coronary microvascular dysfunction (CMD) is one putative mechanism leading to INOCA (4), defined as the spectrum of structural and functional alterations at the level of epicardial, microvascular endothelial that limits myocardial perfusion, which is most often detected as the reduction of coronary flow reserve (CFR) (5, 6). Meanwhile, contemporary studies have suggested that the presence of CMD in INOCA patients further increases the risk of adverse events (7, 8). Nevertheless, the risk factors for CMD in INOCA patients are still unclear.

The effects of abnormal thyroid hormone levels on the cardiovascular system have been well demonstrated (9, 10). Since the widespread presence of thyroid hormone receptors in myocardial tissue, subtle changes in thyroid hormone levels within the reference range can also cause deregulation of the cardiovascular system and have shown a similarly strong connection with cardiovascular risk factors (11). In particular, the higher free thyroxine (FT4)/free triiodothyronine (FT3) ratio reflects the decrease in tissue-specific deiodinase activity and was demonstrated as a novel biological marker to predict the prognosis of several cardiovascular diseases (12, 13), which led us to hypothesize that this ratio would also probably relevant with CMD in euthyroid INOCA patients.

The current gold standard for noninvasive assessment of CFR is positron emission tomography (PET) myocardial absolute blood flow measurement (14, 15). Due to the restrictions in scanning equipment and costs, using dynamic cardiac SPECT (D-SPECT) to estimate CFR has been gradually applied in clinical practice and has been validated to maintain a good agreement with PET (16–18). Therefore, this study was designed as a prospective trial to assess the potential correlation between FT4/FT3 ratio and D-SPECT derived CFR, while determining whether it is an independent risk factor for CMD in euthyroid INOCA patients to further improve the risk stratification.

## Materials and methods

### Study population

**Figure 1:**
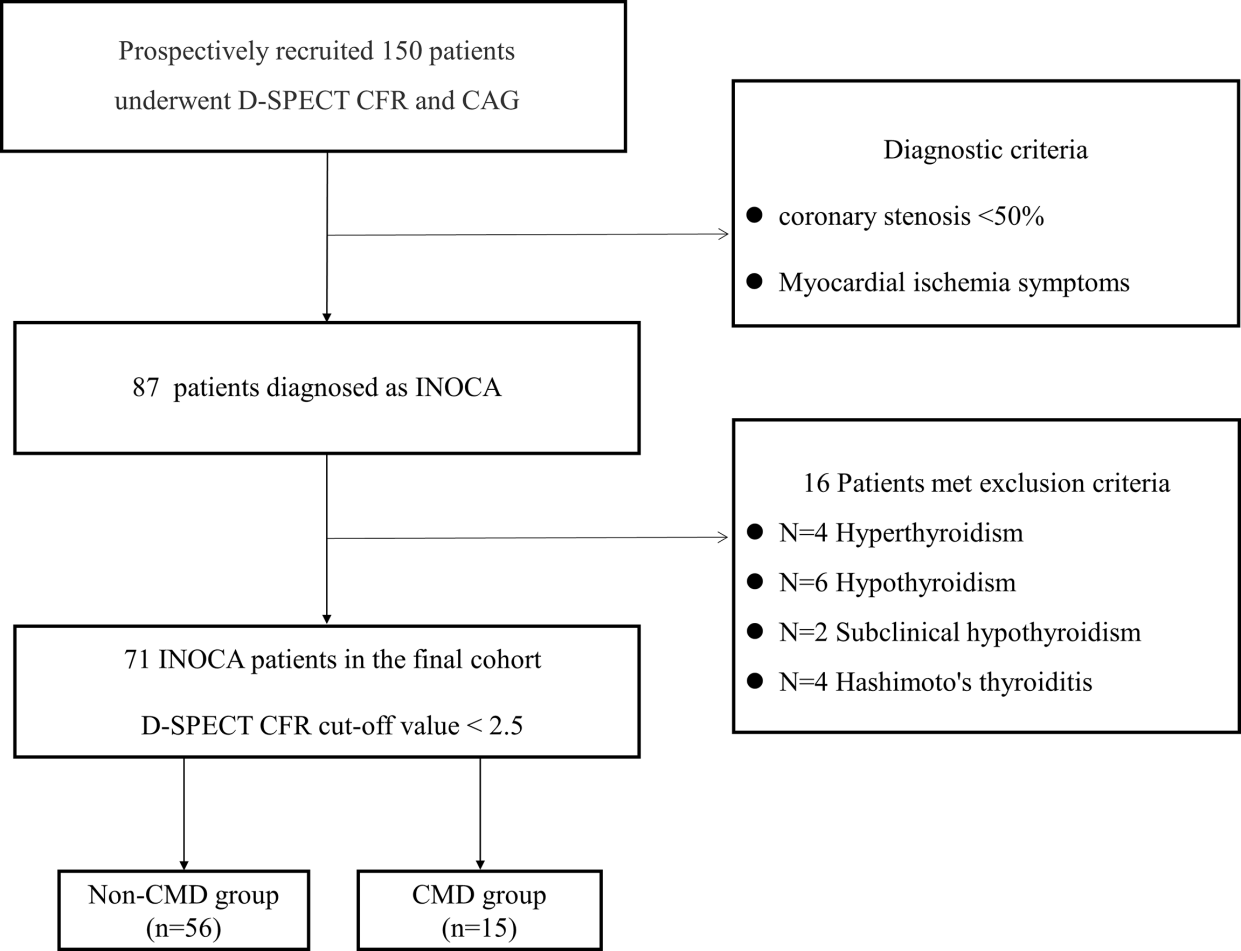
Patient flow chart.

In this prospective study, we recruited 150 patients with myocardial ischemia symptoms who underwent both CAG and D-SPECT CFR within two days in Shanghai Tenth people’s hospital from 2020 to 2021. After excluding the previous history of myocardial infarction or coronary revascularization, 87 patients who present non-obstructive coronary stenosis (<50%) were diagnosed as INOCA. Further biochemical examinations excluded 16 patients: Hyperthyroidism (n=4); Hypothyroidism (n=6); Subclinical hypothyroidism (n=2); Hashimoto’s thyroiditis (n=4). A total of 71 euthyroid INOCA patients were included in the final study cohort. We divided them into two groups (1): the CMD group (CFR<2.5), and (2) non-CMD group (CFR≥2.5). The patient flowchart is shown in **Figure 1**.

### Biochemical assessment

Fasting blood was obtained to test thyroid hormone levels [including free triiodothyronine (FT3), free tetraiodothyronine (FT4), total triiodothyronine (TT3), total tetraiodothyronine (TT4), thyroid stimulating hormone (TSH), thyroglobulin autoantibodies (TGAb), thyroid peroxidase antibody (TPOAb), TSH receptor antibodies (TRAb)].

Thyroid function profile was measured by direct chemiluminescence method (ADVIA Centaur XP, Siemens, USA). The reference intervals were as follows: FT3:2.8-6.3pmol/L, FT4:10.5-24.4pmol/L, TT3:1.0-3.0nmol/L, TT4:55.5-161.3nmol/L, TSH:0.38-4.34pmol/L, TGAb:<110IU/ml, TPOAb:<40IU/ml, TRAb:0-1.75IU/L.

### Dynamic SPECT acquisition

All patients underwent a one-day rest-stress dynamic and conventional acquisition on D-SPECT (Spectrum Dynamics Medical Ltd., Israel, Caesarea, Serial Number: 5217). Stop taking β-blockers, theophylline, dipyridamole, and ACEI drugs for at least 12 h before examination. All scans were performed after overnight fasting.

For rest imaging, an initial dose of approximately 37 MBq (1 mCi) is required to position the patient’s heart within the field of view and to establish the scanning ROI. The remaining dose of approximately 370 MBq (10 mCi) is then injected into the patient, and the rest dynamic images were acquired by list mode for over 6 min. After a 60-90 min delay, rest perfusion scanning was performed.

**Figure 2:**
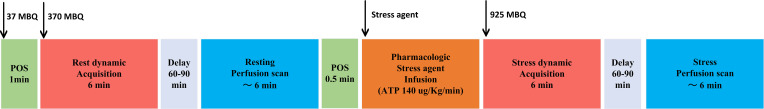
D-SPECT MPI-CFR workflow.

For stress imaging, no positioning dose is required as the heart location can be identified from the resting activity. Pharmacological stress agent ATP was instilled at a rate of 140 ug/(kg·min) through the venous access and 925 MBq (25 mCi) MIBI was injected 3 min after the start of ATP injection. Then stress dynamic images were acquired in list mode over 6 min. After a 60-90 min delay, stress perfusion scanning was performed. The dynamic imaging protocol is illustrated in **Figure 2**.

### Dynamic SPECT analysis

Dynamic imaging data is re-binned into 32 frames consisting of 3 sec x 21 frames, 9 sec x 1 frame, 15 sec x 1 frame, 21 sec x 1 frame, 27 sec x 1 frame, and 30 sec x 7 frames. An OSEM technique is used for the reconstruction of dynamic imaging acquisitions, with 4 iterations and 32 subsets. All dynamic data and corresponding perfusion information are analyzed using semi‐quantitative methods in Corridor 4DM software (INVIA, Ann Arbor, MI, USA). Left ventricular (LV) endocardial and epicardial surfaces are algorithmically estimated from summed myocardial images beyond the two-minute mark. A midwall surface, determined equally distant between the endocardial and epicardial surfaces, is divided into 460 polar map sectors, where LV myocardial tissue time-activity curves (TAC) are the nearest neighbor sampled at the center of each sector across all time frames. The CFR analysis makes use of ROI blood sampling by averaging a box‐shaped region in the LV blood pool, specifically in the center of the LV on the short axis and centered at the basal valve plane along the long-axis, across all time frames. The size of the ROI is two pixels wide on the short axis and 30mm long on the long-axis to sample both the LV and left atrial cavities. The Myocardial blood flow (MBF) was estimated through the previously established net retention model (19) and CFR was calculated as the ratio of the stress MBF to the rest MBF.

### Statistics analysis

Continuous data were presented as mean ± standard deviation, and categorical data were presented as frequency and percentage. The 2 test was used for categorical data. Independent samples t-test was used to compare the means of continuous variables between the CMD group and non-CMD group. The receiver operating characteristic curve (ROC) was performed to calculate the best cut-off value of thyroid hormone for CMD prediction. Pearson’s correlation was used to clarify the relationship between thyroid hormone and CFR. The least absolute shrinkage and selection operator (LASSO) regression was used to select the variables that entered into multivariate logistic regression. Multivariable logistic regression analysis was used to explore the association between FT4/FT3 ratio and CMD while controlling for potential confounders. Results were presented as odds ratio (OR) and 95% confidence interval (95% CI). A two-sided P value of < 0.05 was considered significant. All statistical analysis were performed using SPSS 16.0 for Windows (SPSS Inc., Chicago, IL, USA) and MedCalc 18.0.

## Results

### Baseline characteristics

A total of 71 INOCA patients (mean age 63.2 ± 9.3 years; 36 [50.7%] male) were included in this prospective study. Baseline characteristics, cardiovascular risk factors, and followed medications were compared in **Table 1**. With the CFR cut-off value < 2.5, 15 [21.1%] patients were presented CMD and was significantly older than the non-CMD group (68.6 ± 8.0 vs 61.8 ± 9.1, p=0.01). Meanwhile, the proportion of 2 vessel disease was higher in the CMD group (33.3% vs 10.7%, p=0.04) and no patients with 3 vessel disease in either group.

**Table 1:** Clinical characteristics of the study population (n = 71).

	Total (n=71)	Non-CMD (n=56)	CMD (n=15)	*P* value	
**Patient characteristics**				
Age, years	63.21 ± 9.28	61.77 ± 9.13	68.60 ± 7.98	0.01*
Male gender, n (%)	36 (50.7%)	29 (51.8%)	7 (46.7%)	0.72
Height, cm	167.26 ± 8.81	166.79 ± 9.02	168.86 ± 8.19	0.44
Weight, Kg	71.63 ± 12.05	70.70 ± 12.03	74.86 ± 11.96	0.26
Body mass index, kg/m^2^	25.49 ± 3.28	25.29 ± 3.37	26.15 ± 2.99	0.40
Hypertension, n (%)	45 (63.4%)	33 (58.9%)	12 (80.0%)	0.13
Diabetes, n (%)	10 (14.1%)	9 (16.1%)	1 (6.7%)	0.68
Dyslipidaemia, n (%)	6 (8.5%)	5 (8.9%)	1 (6.7%)	1
Current Smoker, n (%)	13 (18.3%)	11 (19.6%)	2 (13.3%)	0.72
HDL, mmol/L	1.14 ± 0.28	1.14 ± 0.26	1.14 ± 0.34	0.97
LDL, mmol/L	2.47 ± 0.76	2.48 ± 0.80	2.44 ± 0.58	0.86
Cholesterol, mmol/L	4.20 ± 0.87	4.23 ± 0.90	4.12 ± 0.76	0.69
Triglycerides, mmol/L	1.73 ± 1.23	1.78 ± 1.35	1.54 ± 0.68	0.50
Glomerular filtration rate	87.55 ± 15.05	89.16 ± 14.60	82.04 ± 15.83	0.12
**Baseline Medications**
Aspirin, n (%)	22 (31.0%)	16 (28.6%)	6 (40%)	0.53
Statins, n (%)	54 (76.1%)	44 (78.6%)	10 (66.7%)	0.33
Beta-blockers, n (%)	24 (33.8%)	21 (37.5%)	3 (20%)	0.20
CCB, n (%)	20 (28.2%)	14 (25.0%)	6 (40.0%)	0.33
ACEI or ARB, n (%)	20 (28.2%)	17 (30.4%)	3 (20.0%)	0.53
Nitrate, n (%)	8 (11.3%)	6 (10.7%)	2 (13.3%)	0.67
**Angiographic findings**
LAD	40	34	6	
LCX	3	3	0	
RCA	16	8	8	
0-vessel, n (%)	23 (37.7%)	17 (30.3%)	6 (40.0%)	0.63
1-vessel, n (%)	37 (52.1%)	33 (58.9%)	4 (26.7%)	0.009*
2-vessels, n (%)	11 (15.5%)	6 (10.7%)	5 (33.3%)	0.04*
3-vessels, n (%)	0 (0.0%)	0 (0.0%)	0 (0.0%)	

* Represent P < 0.05.

**Figure 3:**
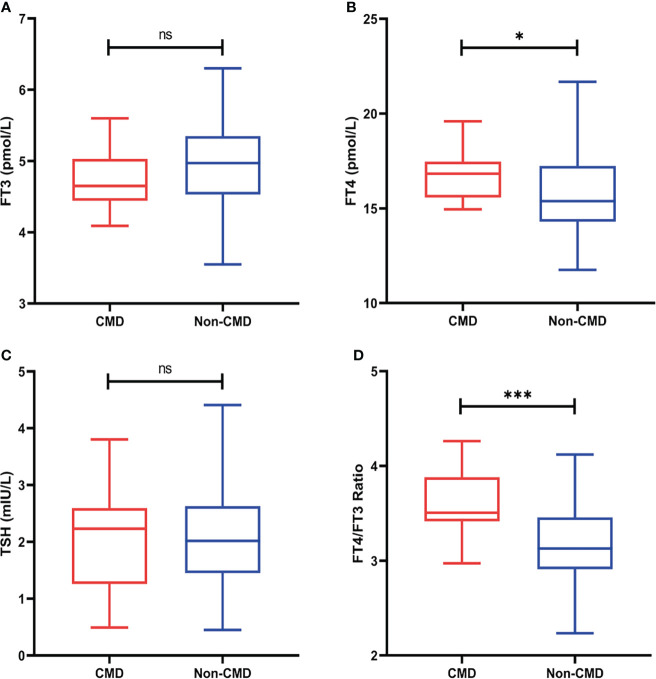
**(A)** FT3 levels between two groups **(B)** FT4 levels between two groups **(C)** TSH levels between two groups **(D)** FT4/FT3 ratio levels between two groups (ns, no significant, *p<0.05, **p<0.01, ***p <0.001).

Thyroid hormone levels, resting/stressing myocardial blood flow, and global CFR was illustrated in **Table 2**. FT4 (16.90 ± 1.36 vs 15.55 ± 2.00, p=0.02) and FT4/FT3 ratio (3.61 ± 0.37 vs 3.16 ± 0.44, p<0.001) were significantly higher in the CMD group. The differences between other thyroid hormones were not statistically significant and were shown in **Figure 3**. As expected, the Stress MBF (2.82 ± 0.95 vs 4.34 ± 1.23, p=0.002) and CFR (2.00 ± 0.34 vs 3.62 ± 0.95, p<0.001) were significantly lower than non-CMD group.

**Table 2:** Thyroid hormone level and CFR of the study population.

	Total (n=71)	Non-CMD (n=56)	CMD (n=15)	*P* value
**Thyroid hormone level**				
FT3, pmol/L	4.90 ± 0.55	4.96 ± 0.58	4.71 ± 0.40	0.13
FT4, pmol/L	15.83 ± 1.95	15.55 ± 2.00	16.90 ± 1.36	0.02*
TT3, nmol/L	1.58 ± 0.27	1.61 ± 0.27	1.49 ± 0.25	0.11
TT4, nmol/L	96.55 ± 17.14	95.09 ± 15.41	101.87 ± 22.20	0.18
TSH, mIU/L	2.13 ± 0.92	2.15 ± 0.90	2.07 ± 1.02	0.85
FT4/FT3 ratio	3.25 ± 0.46	3.16 ± 0.44	3.61 ± 0.37	0.001*
**Myocardial Blood Flow**				
Stress MBF, ml/min/g	4.07 ± 1.31	4.34 ± 1.23	2.82 ± 0.95	0.002*
Rest MBF, ml/min/g	1.29 ± 0.49	1.27 ± 0.50	1.25 ± 0.46	0.54
CFR	3.27 ± 1.08	3.62 ± 0.95	2.00 ± 0.34	<0.001*

* Represent P < 0.05

### Correlation between thyroid hormones and CFR

**Figure 4:**
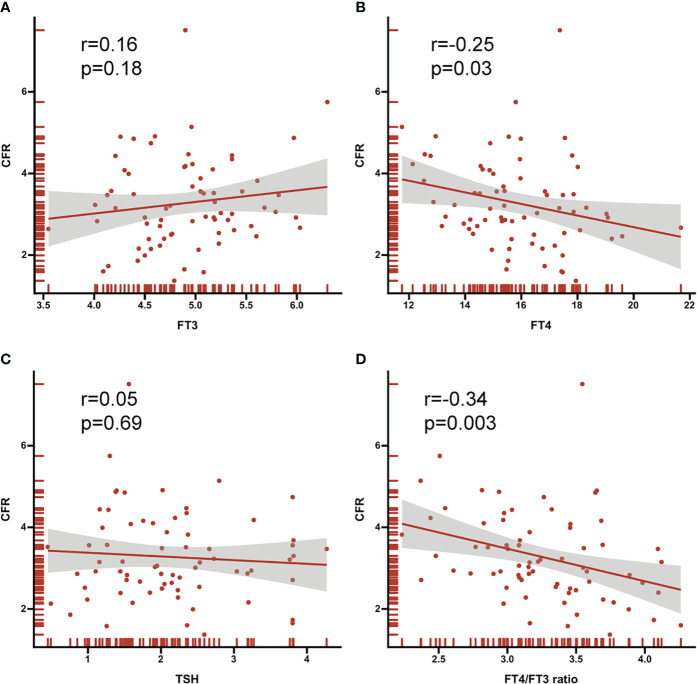
Correlation between thyroid hormone **(A)** FT3 **(B)** FT4 **(C)** TSH **(D)** FT4/FT3 ratio and CFR. Correlation coefficients (r) and P values are shown.

**Figure 5:**
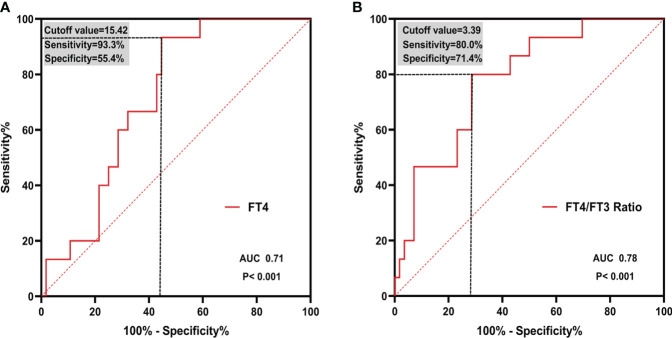
ROC curves of **(A)** FT4 and **(B)** FT4/FT3 ratio in predicting CMD. Cutoff value and corresponding sensitivity and specificity are shown. ROC, receiver operating characteristic; AUC, area under the curve.

As depicted in the **Figure 4**, both FT4 and FT4/FT3 ratio showed a modest significant correlation with CFR (r=-0.25, p=0.03; r=-0.34, p=0.003, respective). ROC curve analysis was used to identify the relative intensity of the association between FT4, FT4/FT3 ratio and CFR. FT4/FT3 ratio had highest AUC 0.78 (95%CI 0.67-0.87, p<0.001), which was larger than that for FT4(AUC 0.71, 95%CI 0.60-0.82, p<0.001). Using Youden’s index to calculate the best cut-off value for FT4/FT3 ratio was >3.39 (sensitivity 80.0% and specificity 71.4%) and FT4 was >15.42 (sensitivity 93.3% and specificity 55.4%). All results are summarized in **Figure 5**.

### Risk factors of CMD

**Figure 6:**
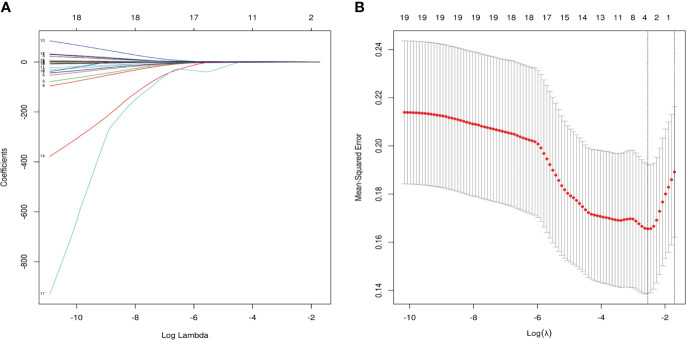
Variables were selected by least absolute shrinkage and selection operator (LASSO) regression. **(A)** Three clinical variables with nonzero coefficients were selected by optimal lambda (λ). **(B)** 10-fold Cross-validation plot for the penalty term. The parameter λ = 0.079 were selected *via* the minimum criteria.

As shown in **Figure 6**, a total of 19 relevant clinical variables (including chronic medical history, thyroid hormones, and cardiovascular-related biochemicals), 3 potential features were selected with nonzero coefficients in the LASSO regression model (FT4, FT4/FT3 ratio and age). After including the above three variables into multivariate regression, the results indicate that FT4/FT3 ratio (OR 7.62, 95% CI 1.12-51.89, p=0.038) and age (OR 1.09, 95% CI 1.00-1.19, p=0.037) still were independent predictors of CMD in INOCA patients. The results of multivariate logistic regression are demonstrated in **Table 3**. The FT4/FT3 ratio and age were further trisection into 3 groups for trend testing. As shown in **Table 4**, with Q1 set as the reference, the FT4/FT3 ratio (OR 14.79, 95% CI 1.69-129.52, P _for trend_=0.006) and age (OR 7.08, 95% CI 1.30-38.44, P _for trend_=0.02) in Q3 were both associated with an increased risk for CMD.

**Table 3:** Multivariate Logistic regression analysis for predicting CMD.

Variables after LASSO regression	OR (95%CI)	*P* value
Age, years	1.09 (1.00-1.19)	0.037*
FT4/FT3 ratio	7.62 (1.12-51.89)	0.038*
FT4	1.22 (0.81-1.85)	0.34

* Represent P < 0.05

**Table 4:** Logistic regression analysis for predicting CMD according to trisection of predictors.

Predictors	CMD				
	No. of cases		OR		95% CI
**FT4/FT3 ratio**
Q1 (<3.080)	24		1.00	
Q2 (3.080-3.456)	24		6.05		0.65-56.36
Q3 (>3.456)	23		14.79		1.69-129.52
***P*_for trend_**			0.006*
**Age (years)**					
Q1 (<59)	25		1.00		
Q2 (59–66)	25		2.87		0.50-16.48
Q3 (>66)	21		7.08		1.30-38.44
***P*_for trend_**			0.02*

* Represent P < 0.05.

## Discussion

Our study has the following three main findings. Firstly, FT4 and FT4/FT3 ratio levels were higher in CMD patients; secondly, both FT4 and FT4/FT3 ratios were correlated with CFR; finally, it was demonstrated that the increased FT4/FT3 ratio predicted the risk of CMD in euthyroid INOCA patients, which provide a novel biological marker to improving risk stratification.

### Baseline characteristic

CMD plays a pivotal role in INOCA patients, which is considered to be the third mechanism causing myocardial ischemia in addition to coronary atherosclerosis and vasospasm (20, 21). Since there is no severe stenosis in the epicardial coronary arteries, the CFR truly reflects the microvascular function of INOCA patients. In our present study, 21.1% of INOCA patients presented with CMD, slightly lower than reported in previous research (22), probably because we excluded some INOCA patients with abnormal thyroid function. Meanwhile, the higher proportion of patients with 2-vessels stenosis also considerably explains the decrease in CFR in the CMD group. Notably, although CMD has been shown to be common complication of type 2 diabetes mellitus (T2DM) (23–27), differences between the two groups did not significantly due to our small sample size. Meanwhile, other traditional cardiovascular risk factors such as hypertension, hyperlipidemia, and smoking in this study also appeared similarly due to the sample size.

### Clinical predictors

Previous studies have established a strong association between abnormal thyroid hormones and increased cardiovascular risk (9). To our knowledge, our study is the first prospective cohort on the relationship between thyroid function in the reference range and CMD risk. Although not a biological hormone directly involved in the regulation of the cardiovascular system, our results suggest that the FT4/FT3 ratio, and not FT4, is an independent risk factor for CMD. Since the affinity of thyroid hormone receptors for T3 is tenfold higher than that of T4, the endocrine system tends to convert T4 to T3 for biological activity, while the FT4/FT3 ratio reflects this dynamic conversion process and is considered the surrogate for evaluating the degree of peripheral thyroxine deiodination and deiodinase activity (9). Similar to our research, recent studies have shown that an elevated FT4/FT3 ratio is associated with adverse prognosis in a variety of cardiovascular diseases.Yuan. et al retrospectively analyzed 3549 euthyroid patients with prior cardiovascular events history undergoing PCI and confirmed that an increased FT4/FT3 ratio predicts all-cause death and cardiac death (28). Meanwhile, for another coronary artery disease in which CMD is involved in the pathogenesis, Gao. et al. found that a lower level of FT3/fT4 ratio remained a robust predictor of MACE in euthyroid MINOCA patients (29). When we focus on non-cardiac disease studies, high levels of FT4/FT4 ratio still represent the worsening illness condition. Maruzzo. et al. performed a prognostic follow-up analysis with 134 mRCC patients, results showed that the low FT3/FT4 ratio independently predict progression-free survival (PFS) and overall survival (OS) (30).

Our results also showed that FT4 was elevated in CMD patients and increased FT4 levels were correlated with decreased CFR. Although T4 requires conversion to T3 to exert its powerful biological effects, increased FT4 levels can still act directly on thyroid hormone receptors in a variety of tissues to promote excessive NO release from endothelial cells, exaggerated coronary vascular tone, and subsequently leading to endothelial dysfunction (10). Similarly, The recently systematic review involving 11 studies with 30,085 patients showed that elevated FT4 levels within the normal range were associated with the incidence of atrial fibrillation (11). Moreover, Anne R. et al. demonstrated that, in addition to an increased risk of atrial fibrillation, high levels of FT4 were also strongly associated with heart failure and mortality (31). Nevertheless, ROC analysis indicated that the FT4/FT3 ratio was the most effective biomarker in predicting CMD, which suggests that we should focus more on the dynamics of thyroid hormone metabolism rather than a particular thyroid hormone.

Another important finding indicated that age was higher in the CMD group and advancing age was significantly associated with the increased CMD risk. These results may be explained by the fact that aging impairs endothelium-mediated coronary vasodilation and angiogenesis, which in turn promotes structural remodeling of the microvasculature and subsequently leads to reduced perfusion of the microcirculation (32, 33). Similar findings have been reported in a cohort with 327 IHD patients and 419 stenosed vessels, the progressive decrease in CFR in both obstructed and non-obstructed coronary arteries was age-related (34). Of note, through altering the phenotype of endothelial cells and smooth muscle cells, microvascular aging also negatively affects the function of the endocrine system in regulating thyroid hormones (35). Strich et al. found that age affects the T4 to the T3 conversion process, older patients are usually accompanied by an elevated FT4/FT3 ratio (36). The CMD group in our research likewise had a higher age and FT4/FT3 ratio, a possible explanation for this might be that increased age leads to decreased metabolism, further leads to reduced deiodinase activity, and more conversion of T4 to reverseT3 (rT3) rather than T3.

### Limitations

This study has several limitations. First, since this study is a single-center prospective study, further sample size expansion and multicenter validation are needed. Second, we require additional follow-up data for this group of patients to clarify the prediction of major adverse cardiovascular events by FT4/FT3 ratio. And some new biological cardiac functional markers associated with thyroid function, such as Brain natriuretic peptide (BNP) (37), could be added in further study to make our results more reliable. Finally, we need to explore the correlation between the FT4/FT3 ratio with different clinical classifications of CMD.

### Conclusion

Taken over, increased FT4/FT3 ratio is strongly consistent with an increased risk of CMD, the high predictive efficacy for CMD is expected to provide a novel biomarker for early prevention and risk stratification in euthyroid INOCA patients.

## Data availability statement

The original contributions presented in the study are included in the article/**Supplementary Material**, further inquiries can be directed to the corresponding author/s.

## Ethics statement

The studies involving human participants were reviewed and approved by Shanghai Tenth people’s hospital ethics committee. The patients/participants provided their written informed consent to participate in this study.

## Author contributions

FY, QH: conception and design. HZ, WC and YH: data collection and analysis. KS: Language editing and polishing. CX, XF, MF, SQ and XH: data curation. JZ, FH and XZ: Provision of study materials. All authors contributed to the article and approved the submitted version.

## Funding

The study was supported by research grants from the National Key Research and Development Program of China (2020YFA0909000), The National Natural Science Foundation of China (82127807), Shanghai Key Laboratory of Molecular Imaging (18DZ2260400), Clinical Research Plan of SHDC (2020CR4065) and The National Natural Science Foundation of China (No.82071956).

## Conflict of interest

The authors declare that the research was conducted in the absence of any commercial or financial relationships that could be construed as a potential conflict of interest.

## Publisher’s note

All claims expressed in this article are solely those of the authors and do not necessarily represent those of their affiliated organizations, or those of the publisher, the editors and the reviewers. Any product that may be evaluated in this article, or claim that may be made by its manufacturer, is not guaranteed or endorsed by the publisher.
