# Supplementary figures and images for: FT4/FT3 ratio: A novel biomarker predicts coronary microvascular dysfunction (CMD) in euthyroid INOCA patients
Source: Front Endocrinol (Lausanne). 2022 Sep 15;13:1021326. doi: 10.3389/fendo.2022.1021326 (PMC9520241; doi:10.3389/fendo.2022.1021326)

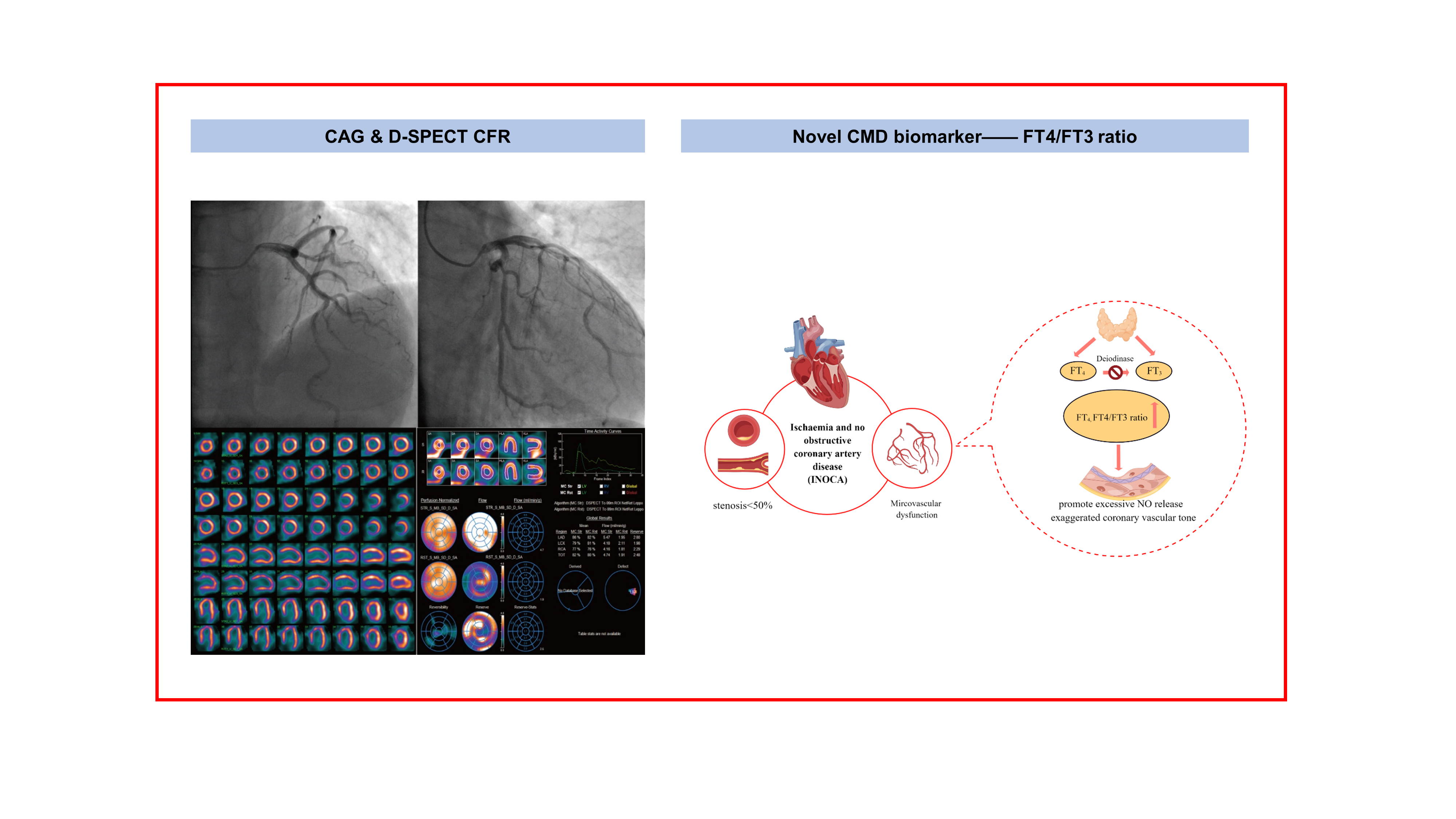

Supplement: Supplementary file 1 [file Image_1.tif]
